# Growth Plate Injury Leading to Madelung‐Type Deformity After ESIN in Children: A Case Report and a Narrative Review of the Literature

**DOI:** 10.1002/ccr3.71890

**Published:** 2026-01-21

**Authors:** Andrea Cosentino, Gianni Odorizzi, Wilhelm Berger

**Affiliations:** ^1^ Department of Orthopedics and Traumatology Hospital of Merano (SABES‐ASDAA) ‐ Lehrkrankenhaus der Medizinischen Privatuniversität Merano‐Meran Italy; ^2^ Paracelsus Medical University Salzburg Austria

**Keywords:** growth plate closure, intramedullary fixation, long‐term follow‐up, pediatric orthopedic complication, physeal injury

## Abstract

Late asymmetric growth arrest of the distal ulna and subsequent Madelung‐type deformity may occur following elastic stable intramedullary nailing (ESIN) performed near the distal ulnar physis. Although causality cannot be definitively proven, meticulous surgical technique and structured long‐term radiographic follow‐up are essential to minimize risk and enable early detection of growth disturbance.

## Graphical abstract

Limitation of the movement 9‐year operated both bone forearm fracture.

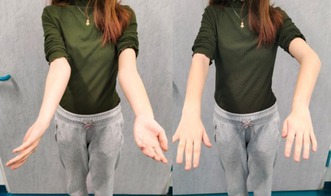

## 1 Introduction

Elastic Stable Intramedullary Nailing (ESIN) is widely recognized as an effective treatment for pediatric forearm fractures. Its minimally invasive technique preserves the periosteal blood supply and typically yields excellent functional outcomes, making it the standard of care for diaphyseal fractures. However, ESIN is sometimes employed for more distal fractures, where precise technique and careful preservation of the growth plate become critically important [1].

Particular challenges arise during the insertion and removal of ESIN in the distal radius and ulna due to the proximity of the entry points to the physis. During insertion, excessive force, poor trajectory planning, or multiple drilling attempts can directly injure or compress the growth plate, increasing the risk of asymmetric physeal arrest. Removal also carries hazards: incomplete metaphyseal ossification in children can make extraction technically demanding, with torsion, leverage, or inadvertent cortical perforation risking subtle physeal damage. These injuries may go unrecognized at the time of surgery but can lead to premature closure and progressive deformity over subsequent years.

The choice of entry point for ulnar nailing is especially important. In anterograde ESIN, the olecranon apophysis is at risk of physeal violation if the entry point is too proximal or misaligned [2]. In retrograde ESIN, the distal metaphyseal entry is very close to the distal ulnar physis, where oblique trajectories or forceful insertion can cause direct growth plate damage [1, 3]. Such iatrogenic injuries can lead to asymmetric growth arrest, a mechanism well documented in cases of posttraumatic Madelung‐type deformity following ESIN [4].

Madelung deformity is characterized by volar and ulnar tilt of the distal radius, dorsal subluxation of the ulna, and resultant pain or functional impairment. While classically associated with congenital conditions such as Leri‐Weill dyschondrosteosis [5], similar deformities can arise post‐traumatically through asymmetric physeal arrest caused by direct injury or surgical trauma [6].

This report describes a 12‐year‐old girl who developed a Madelung‐type wrist deformity several years after ESIN removal for distal forearm fractures, highlighting the potential risk of physeal damage associated with intramedullary nailing and emphasizing the importance of long‐term follow‐up. A narrative review of the literature is included to contextualize this complication.

## 2 Case History/Examination

**FIGURE 1:**
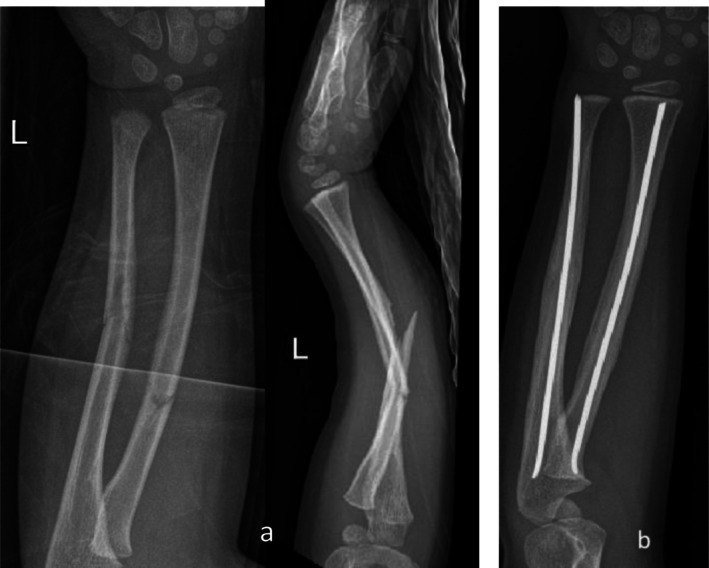
(a) AP and LL view of displaced fracture of the radius and ulna; (b) Postoperative radiograph showing retrograde ESIN fixation and healing.

A previously healthy girl sustained a displaced fracture of the radius and ulna at the age of 4 years and 6 months following a fall from standing height (Figure 1a). She underwent closed reduction and internal fixation using two retrograde elastic stable intramedullary nails (ESIN), one in each bone, using a dorsal entry point for the radius. According to AO surgery reference, for the ulna, a retrograde entry point was created at the distal metaphysis, about 1 cm proximal to the distal ulnar physis. Nail insertion was performed under fluoroscopic guidance to confirm the correct trajectory and to avoid physeal violation. The nail diameter was selected to be about 1/3 of the intramedullary canal size and pre‐bended, ensuring stable fixation without excessive endosteal pressure. The surgery was uneventful, and the fracture alignment was deemed satisfactory on intraoperative fluoroscopy.

Postoperative follow‐up at one month (Figure 1b) demonstrated complete fracture alignment, appropriate healing progression, and no signs of hardware‐related complications. Based on this, the patient was discharged from regular follow‐up. The intramedullary nails were subsequently removed 3 months postoperatively without complication; no technical difficulty during nail removal was reported in the operative notes.

**FIGURE 2:**
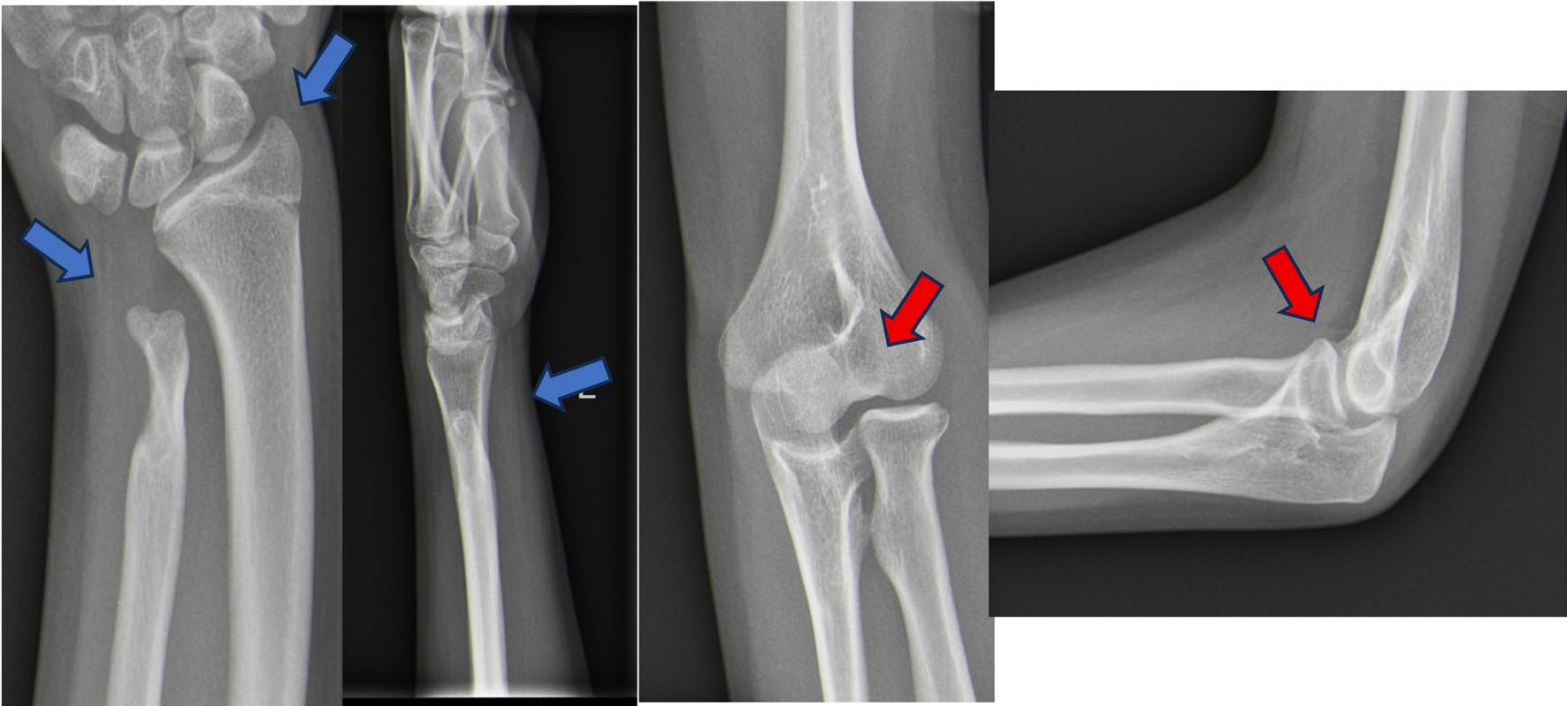
Anteroposterior and lateral radiographs of the wrist demonstrating a Madelung‐type deformity with increased volar tilt of the distal radius and negative ulnar variance (red arrows). The elbow shows the subluxation of the proximal radioulnar joint (blue arrows).

Approximately 9 years after the initial injury and hardware removal, the patient presented to our emergency department following a minor elbow contusion during sports activity. Radiographs of the elbow unexpectedly revealed a mild humeral‐ulnar subluxation with an unusual aspect of the proximal ulna. These findings prompted a radiographic evaluation of the wrist, which demonstrated a volar tilt of the distal radius, dorsal prominence of the distal ulna, and increased ulnar variance consistent with a Madelung‐type deformity (Figure 2).

Radiographically, the deformity was characterized by increased volar tilt of the distal radius, negative ulnar variance, and dorsal prominence of the distal ulna, consistent with a Madelung‐type deformity. Comparison with the contralateral wrist demonstrated clear asymmetry in distal radial alignment and ulnar variance. Although formal quantitative measurements at the time of diagnosis were limited by the retrospective nature of imaging availability, the side‐to‐side comparison supported the presence of a clinically relevant growth disturbance.

The patient reported no other wrist trauma or complaints, and the deformity had gone undetected during the interim.

**FIGURE 3:**
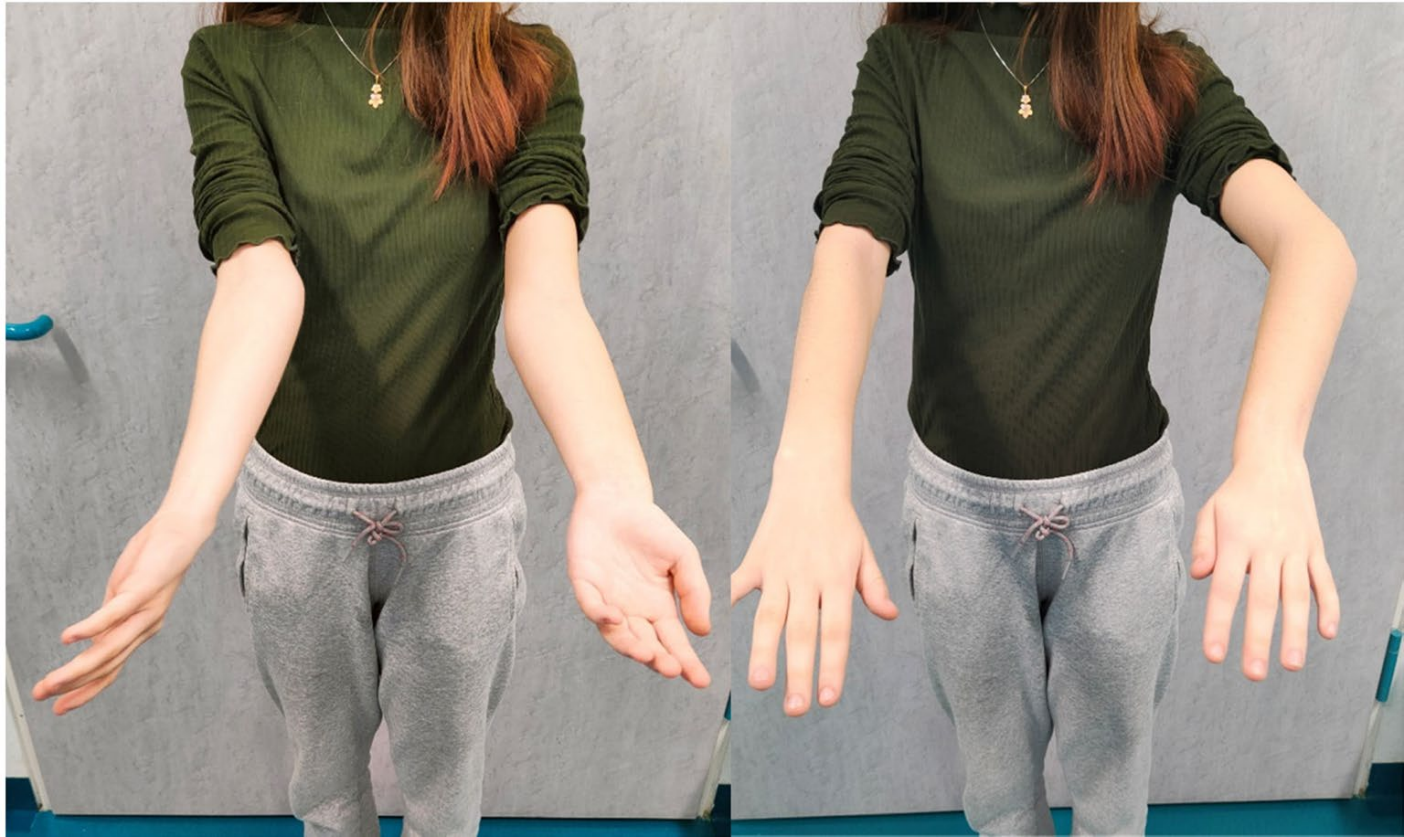
Clinical photographs showing limitation of forearm supination (left) and pronation (right).

Following radiographic diagnosis of the Madelung‐type deformity, the patient was referred for specialist evaluation and comprehensive diagnostic assessment. Clinical examination confirmed limitation of forearm rotation and activity‐related wrist discomfort (Figure 3), without neurovascular compromise. Given the patient's remaining growth potential and the progressive nature of the deformity, conservative management was not considered appropriate.

The case was therefore discussed within a multidisciplinary orthopedic and hand surgeons' team, and surgical treatment options were analyzed, including corrective osteotomy and procedures aimed at restoring distal radioulnar joint congruence. Advanced imaging and preoperative planning were undertaken to define the extent of deformity and guide surgical decision‐making.

## 3 Methods

A narrative review of the literature was conducted to identify relevant studies on posttraumatic or iatrogenic Madelung‐type deformity following pediatric forearm fracture treatment, particularly with ESIN.

### 3.1 Search Strategy

We performed a comprehensive search of the PubMed database from inception through June 2025. The following keywords were used: (“Madelung deformity” OR “Madelung‐type deformity”) AND (“ESIN” OR “elastic stable intramedullary nailing”) AND (“pediatric” OR “child” OR “adolescent”) AND (“ulna” OR “forearm fracture”). Additional articles were identified by manually screening the reference lists of included papers.

Studies were eligible if they reported cases of Madelung‐type deformity or wrist deformities following pediatric forearm fracture fixation, included patients under 18 years of age, described the use of ESIN or comparable intramedullary fixation methods, and provided clinical or radiological documentation of growth disturbance or deformity.

We excluded studies not in English, papers without full‐text availability, and studies focused solely on congenital or syndromic Madelung‐type deformity without a posttraumatic or surgical etiology.

ASReview was used as a supportive tool to assist literature identification.

### 3.2 Data Extraction and Analysis

Data extraction focused on identifying studies that described pediatric patients (< 18 years) treated for forearm fractures with ESIN and that reported complications relevant to growth disturbance, deformity, or physeal injury, particularly Madelung‐type deformities.

Due to the small number of eligible studies and their clinical heterogeneity (including case reports and retrospective series with variable reporting detail), we conducted a descriptive qualitative synthesis. Key study characteristics and findings were summarized in tabular form (Table 1), and narrative synthesis was used to highlight shared themes, patterns of complication, and implications for clinical practice.

**TABLE 1:** Summary of included studies.

First author (Year)	Study type	Population	Key focus	Findings	References
Muhammad (2023)	Case Report	Pediatric patient	Posttraumatic Madelung deformity after ESIN	Describes distal ulnar physeal arrest leading to classic Madelung deformity; emphasizes need for surgical caution and early detection.	[4]
Kozin and Zlotolow (2015)	Review Article	Mixed pediatric cases	Madelung deformity pathophysiology	Defines congenital vs. acquired forms; highlights trauma and surgical iatrogenic physeal damage as causes of Madelung‐type deformity.	[5]
Ali et al. (2015)	Review	Pediatric imaging studies	Clinical/radiologic features of Madelung	Explores acquired Madelung‐type deformity; notes asymmetrical physeal arrest from trauma or surgery as a mechanism.	[6]
Capitain (2017)	Retrospective Thesis	Pediatric radius fractures	ESIN technique and outcomes	Reviews ESIN use in children; reports low early complications but stresses need for longer‐term monitoring for growth disturbances.	[1]
Du et al. (2016)	Retrospective Cohort	88 pediatric patients	ESIN vs. plate comparison	Compared outcomes for forearm fracture fixation; documented residual deformity and loss of rotation as possible ESIN complications.	[7]
Kruppa et al. (2017)	Retrospective Cohort	45 pediatric patients	ESIN complication rates	Reported overall low complication rates but included angular malunion; underscored need for technique refinement and follow‐up.	[8]

## 4 Discussion and Results of Narrative Review

This case describes the late development of a Madelung‐type deformity in a pediatric patient several years after treatment of a distal forearm fracture with ESIN. Although the temporal sequence raises concern for a possible association between ESIN performed near the distal ulnar physis and subsequent asymmetric growth disturbance, a direct causal relationship cannot be definitively established. No intraoperative or early postoperative imaging documented physeal violation, and the deformity was detected only years later, leaving a substantial temporal gap. Several alternative mechanisms must therefore be considered. An occult physeal injury related to the initial trauma, including an unrecognized Salter–Harris lesion of the distal ulna, cannot be excluded. In addition, a combined mechanism, in which a subclinical traumatic physeal injury is exacerbated by surgical manipulation during nail insertion or removal, may be plausible. These factors highlight the difficulty of attributing late physeal arrest to a single causative event in the absence of contemporaneous physeal imaging. While ESIN is widely regarded as the gold standard for pediatric diaphyseal forearm fractures due to its minimally invasive approach, reliable healing rates, and low profile of complications, physeal‐related deformities remain under‐recognized in the literature.

Our review identified six studies directly relevant to this phenomenon, underscoring both the potential risks of ESIN near the physis and the challenges in detecting late‐onset deformities.

Muhammad and Rukmoyo [4] presented a strikingly similar case of posttraumatic Madelung‐type deformity in a pediatric patient caused by growth arrest of the distal ulnar physis following fracture fixation. Their case reinforces our observation that even with technically successful ESIN, the physis remains vulnerable to iatrogenic injury during nail insertion or removal. Both cases highlight the subtlety with which such deformities may progress over years, often asymptomatic until adolescence, when growth imbalance becomes pronounced.

Ali et al. [6] and Kozin and Zlotolow [5] both emphasize that Madelung‐type deformities can arise not only from congenital or syndromic causes but also from asymmetric growth arrest due to trauma or iatrogenic injury. These reviews explain that even minimal physeal damage to the distal ulna can lead to tethering of growth on the volar‐ulnar side, with the radius continuing unbalanced growth. This mechanism explains the characteristic radiographic features we observed, including increased volar tilt and positive ulnar variance.

Capitain [1] reviewed ESIN fixation in pediatric radius fractures, noting that while ESIN has a low complication rate, there is potential for unmonitored deformity progression in the absence of scheduled follow‐up.

Our case underlines this point: despite perfect alignment at early follow‐up, the patient was lost to routine surveillance after hardware removal, with the deformity only detected incidentally years later. This suggests that routine follow‐up beyond fracture union may be advisable in cases with distal physeal proximity.

Broader case series on pediatric ESIN, such as those by Du et al. [7] and Kruppa et al. [8], report generally low overall complication rates but do document cases of malunion, angular deformity, and loss of rotational alignment. While these studies did not specifically report Madelung‐type deformity, they support the notion that even small residual angulations or unnoticed physeal injuries can have long‐term remodeling consequences in growing children.

Our review underscores the need for:

- Meticulous surgical technique to avoid distal ulnar physeal damage during ESIN insertion and removal.
- Careful timing of nail removal, with awareness of growth plate status.
- Structured long‐term follow‐up that extends beyond initial fracture union to detect evolving deformity, particularly in children approaching skeletal maturity.

Furthermore, patient and caregiver education regarding potential growth disturbances is essential to ensure early recognition and timely referral if deformity develops.

## 5 Conclusion

This case report and narrative review describe a rare late‐onset Madelung‐type deformity detected years after ESIN fixation of a pediatric forearm fracture. While a direct causal relationship between ESIN and distal ulnar physeal arrest cannot be definitively established, the case highlights a possible association between intramedullary fixation performed near the distal ulnar physis and subsequent asymmetric growth disturbance. Surgeons should exercise meticulous technical care when operating near the physis and maintain long‐term clinical and radiographic follow‐up, as growth‐related complications may remain clinically silent for years before becoming apparent.

## Author Contributions

**Andrea Cosentino:** conceptualization, data curation, methodology, writing – original draft, writing – review and editing. **Gianni Odorizzi:** conceptualization, project administration, validation. **Wilhelm Berger:** data curation, methodology, resources, visualization, writing – review and editing.

## Funding

The authors have nothing to report.

## Ethics Statement

This study was conducted in accordance with the ethical standards of the institutional and national research committee and with the 1964 Declaration of Helsinki and its later amendments. In accordance with institutional policy, formal ethics committee approval was not required for this case report.

## Consent

The patient’s parents signed the informed consent for the operations.

## Conflicts of Interest

The authors declare no conflicts of interest.

## Data Availability

The authors have nothing to report.

## References

[1] A. N. Capitain , Elastic Stable Intramedullary Nail Fixation for Displaced Fractures of the Neck of the Radius in Children (University of Split School of Medicine, 2017), https://repozitorij.mefst.unist.hr/en/islandora/object/mefst:474.

[2] J. M. Flynn and P. M. Waters , Rockwood and Wilkins' Fractures in Children, 8th ed. (Lippincott Williams & Wilkins, 2014).

[3] P. Lascombes , T. Haumont , and P. Journeau , “Elastic Stable Intramedullary Nailing (ESIN) in Children,” Injury 41 (2010): S28–S34.

[4] H. Muhammad and T. Rukmoyo , “Management of Pediatric Madelung's Deformity of the Forearm due to Physeal Growth Arrest of the Distal Ulna: A Case Report,” Annals of Medicine and Surgery 85 (2023): 64–67. 37113878 10.1097/MS9.0000000000000252 PMC10129241

[5] S. H. Kozin and D. A. Zlotolow , “Madelung Deformity,” Journal of Hand Surgery American 40 (2015): 1436–1443. 10.1016/j.jhsa.2015.03.033 26341718

[6] S. Ali , H. Patel , and J. Skirko , “Madelung Deformity and Madelung‐Type Deformities: A Review of the Clinical and Radiological Characteristics,” Pediatric Radiology 45 (2015): 1271–1278. 26135644 10.1007/s00247-015-3390-0

[7] S. H. Du , Y. Z. Feng , Y. X. Huang , et al., “Comparison of Pediatric Forearm Fracture Fixation: Elastic Stable Intramedullary Nailing vs Plate Fixation,” American Journal of Therapeutics 23 (2016): e730–e736. 24413367 10.1097/MJT.0000000000000031

[8] C. Kruppa , P. Bunge , T. A. Schildhauer , et al., “Low Complication Rate of Elastic Stable Intramedullary Nailing in Pediatric Forearm Shaft Fractures,” Medicine (Baltimore) 96 (2017): e6669. 28422876 10.1097/MD.0000000000006669 PMC5406092

